# Announcing changes to the publishing procedures of “Biophysics and Physicobiology” (BPPB)—the Biophysical Society of Japan’s English language biophysics journal

**DOI:** 10.1007/s12551-021-00882-x

**Published:** 2021-11-16

**Authors:** Haruki Nakamura

**Affiliations:** Osaka University, Suita, Osaka 565-0871 Japan

## Abstract

This Commentary describes some upcoming changes to the submission and payment procedures to the Biophysical Society of Japan’s English language journal “Biophysics and Physicobiology” (BPPB) that will facilitate a much easier and cheaper publishing experience for all scientists—whether they be Japan-based or located internationally.

The Biophysical Society of Japan (BSJ) runs two publishing vehicles for biophysical content, the Japanese language “Seibutsu Butsuri” and the English language “Biophysics and Physicobiology” (BPPB) (BPPB 2021a). The latter is an on-line open journal with the license reference BY-NC-SA 4.0 (Creative Commons 2021). This journal inherited all the assets of “BIOPHYSICS,” the former BSJ official journal that had originally been published since 2005, and expanded its scope by changing the journal name in 2015 to keep abreast of future directions in the field. Both official journals use the J-STAGE, which is a governmental electronic journal platform in Japan (J-STAGE 2021).

From October 2021, the publishing procedures of the BPPB journal have been altered to facilitate more rapid and cheaper publication of each accepted article than before. The full details can be found in the updated “Instruction for Authors” on the web page of the BPPB (BPPB 2021b) and only the outline is presented here. The new procedure is as follows:

- Authors will be asked to submit their manuscript using a formatting template that can be downloaded from the web page of “Instruction for Authors” (BPPB 2021b).
- A manuscript transfer system (B2J) from bioRxiv to the BPPB allows authors to save time by transmitting their manuscript files and metadata directly from bioRxiv to the BPPB (more can be read about this at (BioRxiv 2021)).
- The BPPB submission site which uses the S1M (ScholarOne Manuscripts) system (ScholarOne Manuscript 2021) will automatically convert their submission data to the PDF file. In the event that the converted PDF file is incorrect, the authors are requested to provide their own manuscript PDF file.
- After the usual multiple rounds of scientific peer review and revision, all accepted manuscripts will be sent to the BPPB publication office which will then add related official information to the accepted PDF file (such as the new DOI of the article, and the received and accepted dates). Then, the manuscript will be published on-line within a week using the service of “Advance Online Publication” by the J-STAGE.
- The manuscript, which appears as the Advance Online Publication, is examined by the authors with a request for page proofing. During this final copy editing/page proofing stage, the authors are requested only to confirm the PDF file with typically only a single round for changes to be made by the publication office.

The above procedures will allow BPPB to greatly reduce the publication time and cost. The use of a template will, however, lead to a change in the style of articles published in BPPB as follows:

1. Double column setting is changed to single column setting
2. The bibliographic style of the Reference list is slightly changed without using “italic” or “bold-face” fonts.
3. From Volume 19 onwards (published in 2022), each article will be identified by the newly introduced “Article ID,” not by page numbers.
4. The E-mail address of the corresponding author will not be the main identifier. Instead, the ORCID iD (ORCID 2021) will be the principal designator.
5. Authors must submit a “Graphical Abstract” except for articles in the category of “Editorial” or “Commentary and Perspective.”

The BPPB Editorial Board Members hope that such simpler and cheaper to the author systems will allow BPPB to continue to attract a broad range of biophysical content submissions, both from nationally based and internationally based researchers. We encourage you to take a look at the journal and the exciting range of original and reviewed content that it offers (BPPB 2021c).
